# Pathological changes of liver one year later in CHB patients with negative HBV DNA

**DOI:** 10.1186/s13027-019-0265-2

**Published:** 2019-12-10

**Authors:** Wu Shanshan, Du Xinfang, Yu Shuihong, Lai Kecong, Qi Jinjin, Chen Zhi, Chen Feng

**Affiliations:** 1 State Key Laboratory for Diagnosis and Treatment of Infectious Diseases, First Affiliated Hospital, School of Medicine, Zhejiang University, Zhejiang, 310003 Hangzhou China; 2 Beilun People’s Hospital, Ningbo, 315800 China; 3 Beilun Second People’s Hospital, Ningbo, 315809 China

**Keywords:** Hepatitis B, HBV DNA, Coagulation function, Liver function, Blood routine, Pathological analysis

## Abstract

**Background:**

In this study, we aim to determine the hepatic pathological changes in HBV DNA-negative chronic Hepatitis B (CHB) patients after 12-month antiviral therapy.

**Methods:**

Blood routine indicators including platelet count (PLT) and white blood cell (WBC) were determined. The coagulation function was evaluated by determining the prothrombin time (PT) and prothrombin time activity (PTA), together with the HBV DNA quantification and alpha fetoprotein (AFP). The virology data included hepatitis B surface antigen (HBsAg)/antibodies against hepatitis B surface antigen (anti-HBs), hepatitis B e antigen (HBeAg)/antibodies against hepatitis B e antigen (anti-HBe) and antibodies against hepatitis B core antigen (anti-HBc) were tested. Pathological assay was performed to the liver puncture tissues. Based on the HBV DNA data in the 12-month follow-up of the cases that received anti-viral therapy during this time, the experimental group was divided into group A (HBV DNA negative at the baseline level, HBV DNA negative after 12 months, *N* = 79) and group B (HBV DNA negative at the baseline level, HBV DNA turning to be positive after 12 months, *N* = 13). Statistical analysis was performed on the each test index of the two groups.

**Results:**

The inflammation grade of group A showed significant improvement after 12-month treatment (*P* < 0.05). The pathological inflammation grade of group B was increased after one year, and the liver function indices and the PTA (*P* < 0.05) levels were all increased. Pathological results indicated that the proportion of disease progression in group A was decreased after 12-month follow-up while that proportion was increased in group B. Significant differences were noticed in AFP levels between the patients with progression in group A and those with progression in group B.

**Conclusion:**

Negative HBV DNA does not mean a controlled hepatitis B. Hepatitis B patients transferred to HBV DNA positivity during the anti-viral therapy are easily to show disease progression, and then special attention should be paid to the HBV DNA monitoring. Meanwhile, close monitoring to the changes of liver function, PTA and AFP levels may help to detect changes on the disease in a timely manner.

## Background

Hepatitis B virus (HBV) induced chronic hepatitis B (CHB) is mainly characterized by liver inflammation that causes multi-organ damages. Despite the extensive application of hepatitis B vaccine, a large number of patients are suffering from hepatitis B infection in China mainland, and the cure rate is extremely low. HBV, a member of hepadnavirus family, can trigger hepatic inflammation gradually, and then results in fibrosis, cirrhosis and even accounting for at least 50% of the cases with primary hepatocellular carcinoma worldwide [1–4]. Chronic HBV infection is the most important risk factors for HCC [4, 5]. It has been well acknowledged that persistent HBV replication is associated with cirrhosis development, as a consequence, it can lead to liver decompensation and the occurrence of HCC [6]. In clinical settings, nucleoside analogues (NAs) have been commonly utilized for the treating CHB, however, only functional cure is achieved in most cases. In China, the subsequent hazard caused by HBV infection is still a severe threat to the individuals. Nowadays, a great challenge is generated in the public health program in China. With the advances of medical technology and laboratory test technique, extensive efforts have been given to identify indicators that are more specific and sensitive for the diagnosis of hepatitis B.

HBV DNA, an important indicator for HBV replication, is the most direct, specific and sensitive index for the determination of HBV infection [6]. HBV itself causes no damages to hepatocytes directly, but the level of HBV DNA can be utilized to evaluate the replication, infectivity and treatment efficiency of HBV. To date, treatment efficiency in patients with HBV infection is mainly determined by the decline rate, magnitude or negative conversion of HBV DNA titer no matter which kind of antiviral drug treatment is used [7, 8]. HBV DNA titer is an important predictor for the efficacy of antiviral therapy, which contributes to the monitoring and efficacy evaluation after antiviral therapy. Studies confirmed that HBV DNA positive hepatitis B patients showed better prognosis after active treatment [9, 10]. Nowadays, there are still some controversies about the treatment efficiency of antiviral regimen for HBV DNA-negative hepatitis B patients. To understand the correlation between the change of HBV DNA after antiviral therapy and liver disease prognosis in patients with HBV DNA-negative hepatitis B, we designed such retrospective analysis involving the HBV DNA-negative hepatitis B patients who have been receiving antiviral therapy over 6 months. We hope to provide a theoretical basis for deep understanding for the clinical treatment of HBV infection and the development of liver diseases.

## Materials and methods

### Patients

Ninety-two HBV DNA-negative hepatitis B patients (male: 66; female: 26; mean age: 43.50 ± 10.81 yrs) received antiviral therapy for more than 6 months in Beilun People’s Hospital (Ningbo, China) from January 2011 to December 2015 were enrolled in this study. Upon enrollment, the patients continued to receive anti-rival therapy. CHB diagnosis was performed based on the consensus recommendations of the Asian Pacific Association for the study of the liver (APASL) [11]. HBV DNA-negativity was considered as a detection limit of < 1000 IU/ml. The exclusion criteria were as follows: (i) those with other liver diseases, such as drug-induced hepatitis, alcoholic hepatitis, autoimmune hepatitis, or those with liver injuries caused by toxic substances or other causes; (ii) those infected with other hepatitis viruses (e.g. hepatitis A, C, D, E virus) and HIV infection; (iii) patients received medications that may affect immune function within 6 months; (iv) those received radiation or chemotherapy. All participants signed the informed consent. This study was approved by the Ethics Committee of Beilun People’s Hospital (Ningbo, China).

### Grouping

In total, 92 CHB patients underwent antiviral therapy using Entecavir (Zhengda Tianqing Pharmaceutical, Jiangsu, China) or Lamivudine (Glaxosmithkline pharmaceuticals, Jiangsu, China) via oral administration (0.5 mg or 0.1 g, once per day) every morning prior to food and/or drinking for more than 6 months were enrolled in this retrospective study. Persistent anti-viral therapy was given during the 12 months follow-up. HBV DNA quantification was performed following the manufacturer’s instruction (Zhijiang Biotech, Shanghai, China). Real-Time quantitative PCR was used to determine serum HBV DNA using an ABI-7500 system. The detection limit was 1000 IU /ml. Based on the HBV DNA concentration after 12-month antiviral therapy, the patients were classified into group A (HBV DNA negative at the baseline level, HBV DNA negative after 12 months, *N* = 79) and group B (HBV DNA negative at the baseline level, HBV DNA transferred to positivity after 12 months, *N* = 13), respectively.

### Observation indices

Venous blood samples were obtained from each patient and were immediately stored at − 80 °C for subsequent analysis. Liver function was evaluated by determining the levels of aspartate aminotransferase (AST), alanine aminotransferase (ALT), direct bilirubin (DBIL), total bilirubin (TBIL), albumin (ALB), alkaline phosphatase (ALP), globulin (GLB), and glutamyl transpeptidase (γ-GT), using a Hitachi automatic biochemical analyzer (Hitachi7600–110, Japan). Coagulation function was evaluated by determining the level of prothrombin time (PT) and prothrombin activity (PTA). The coagulation function was measured using an automatic coagulation analyzer (Sysmex CS5100, Japan). The blood routine test involved platelet (PLT) count and white blood cell (WBC) that were tested by automatic blood cell analyzer (Sysmex XN, Japan). The hepatic tumor marker determined was alpha-fetoprotein (AFP) and the chemoluminescence microparticle immunoassay (Abbott i2000SR, USA) was used to detect serum AFP in hepatitis B patients. Qualitative detection of virological data included Hepatitis B surface antigen (HBsAg), antibodies against hepatitis B surface antigen (anti-HBs), Hepatitis B e antigen (HBeAg), antibodies against hepatitis B e antigen (anti-HBe), antibodies against hepatitis B core antigen (anti-HBc) determined by Enzyme linked immunosorbent assay (ELISA, Xinchuang, Xiameng, China). Twelve months after Lamivudine or Entecavir administration, we then obtained the serum samples again for the determination of the same indices.

### Pathological analysis

Hepatic tissues were obtained after puncture using 16 G disposable needles (C. R. Bard, NJ, USA). Subsequently, the hepatic biopsy specimens were fixated with 4% paraformaldehyde immediately, and were embedded by using paraffin. The sections were conventionally stained with HE staining. The images were observed under a microscope (Olympus BX51, Tokyo, Japan) for pathological and histological analysis. All liver biopsies in the study were interpreted by the same pathologist. The inflammatory activity or fibrosis in hepatic tissues of chronic hepatitis was divided into 4 grades or stages (i.e. G1–4 or S1–4) in clinical pathological diagnosis, G0 (no hepatic necroinflammation) and S0 (no fibrosis) according to the Scheuer scoring system. A score of 1–4 was utilized in the statistical analysis to indicate the corresponding grade or stage. For the diagnostic criteria of pathological transformation, a stable condition was defined in the patients presenting stable symptoms and physical signs, stability of various serum targets utilized for the liver disease diagnosis, and stable pathological inflammation or fibrosis grade after the 12-month follow-up, especially those with a similar pathological inflammation grade and inflammatory staging. Improved condition was defined as significant remission in the symptoms, physical signs, improvement of various serum targets, pathological inflammation or fibrosis grade in the 12-month follow-up than the baseline levels, especially those with improvement in the pathological inflammation grade and inflammatory staging. Progression was defined as no significant improvement or even deterioration of clinical symptoms, physical and signs, various test findings, and pathological inflammation or fibrosis grade in the 12-month follow-up than the baseline levels, especially those with deterioration in the pathological inflammation grade or inflammatory staging.

### Statistical analysis

SPSS 19.0 software was used for the data analysis. The data were presented as mean ± standard deviation (SD). Differences between two groups were conducted using paired T test or sign rank test. Analysis of variance was carried out on the comparison of more than two groups. Mann-Whitney U test was utilized to evaluate treatment effects. Chi square test or Fisher’s exact test was utilized for the rate comparison. A *P* value of less than 0.05 was considered as statistical significance.

## Results

### Patients characteristics

Among the 92 HBV DNA-negative hepatitis B patients, 79 (85.87%) were still HBV DNA negative 12 months after anti-viral treatment (termed as group A; mean age: 43.57 ± 11.32 years). The resting 13 (14.13%) were HBV DNA positive after 12-months treatment using Entecavir or Lamivudine (termed as group B; average age: 43.08 ± 7.27 years). No statistically significant differences were observed in the age and gender between the two groups (*P* > 0.05, Table 1).

**Table 1:** Patient characteristics before and 12-month after antiviral therapy

Category	Number	Male/female (cases)	Average age
One year ago			
HBV-DNA negative hepatitis B patients	92	66/26	43.50 ± 10.81
One year later			
Group A			
HBV-DNA negative hepatitis B patients	79	59/20	43.57 ± 11.32
Group B			
HBV-DNA positive hepatitis B patients	13	7/6	43.08 ± 7.27

### Comparison of serum targets before and after one year

Twelve months after anti-viral treatment, the serum targets in group A were stable at the same level as the baseline levels. The average level of ALT and viral indices (especially the proportion of HBeAg positive) decreased despite the fact that no statistical difference was observed (*P* > 0.05). AFP, ALB, PT levels in group A showed significant decrease compared with the baseline levels (*P* < 0.05). PTA showed significant increase (*P* < 0.05) compared with the baseline level. The serological results of group B indicated that the indicators of liver function were all increased about 12 months after treatment, especially ALT and AST. Additionally, PTA and AFP showed increase compared with the baseline levels. There were significant differences in the PTA level in the 12-month follow-up when comparing with the baseline level (*P* < 0.05). The TBIL and DBIL levels between group A were significantly different from those of group B at the baseline levels (*P* < 0.05). The ALT and DBIL levels between group A and group B showed significant differences (*P* < 0.05) about 12 months after treatment. Taken together, the inflammatory activity in the liver tissues of hepatitis B patients whose HBV DNA transformed into positivity 12 months after antiviral therapy was more prominent than those were HBV DNA negative (Table 2).

**Table 2:** Comparison of serological indices, inflammation grade and fibrosis grade in HBV DNA negative and positive hepatitis B patients after 12-month follow up

Test Index	Group A		Group B	
	Baseline level	12 months later	Baseline level	12 months later
ALT(U/L)	37.77 ± 41.74	30.21 ± 32.37	40.77 ± 34.72	51.08 ± 47.36^@^
AST(U/L)	30.41 ± 20.97	31.74 ± 35.14	33.92 ± 17.17	43.92 ± 35.53
TBIL (μmol/L)	14.73 ± 6.09	14.78 ± 5.98	10.92 ± 2.76^#^	11.55 ± 3.65
DBIL (μmol/L)	4.70 ± 4.69	4.53 ± 1.98	2.90 ± 1.11^#^	3.25 ± 1.30^@^
ALB(g/L)	47.71 ± 3.66	46.22 ± 3.12*	46.37 ± 5.10	44.58 ± 6.14
GLB(g/L)	27.49 ± 4.51	28.17 ± 4.17	27.07 ± 4.86	28.79 ± 4.98
ALP(U/L)	71.09 ± 23.62	67.75 ± 18.85	67.54 ± 23.49	69.31 ± 23.36
γ-GT (U/L)	30.68 ± 29.56	29.34 ± 23.59	24.08 ± 20.56	26.92 ± 21.25
PT(s)	11.83 ± 0.92	11.23 ± 0.89*	11.29 ± 0.82	10.95 ± 0.92
PTA(%)	94.34 ± 10.15	107.89 ± 9.58*	98.90 ± 8.64	113.06 ± 16.98^&^
WBC(10E9/L)	5.36 ± 1.36	5.87 ± 1.78	6.18 ± 1.82	6.60 ± 2.43
PLT(10E9/L)	158.68 ± 55.02	159.78 ± 45.61	170.33 ± 48.66	168.62 ± 36.71
AFP (ng/mL)	4.05 ± 2.94	2.85 ± 1.63*	3.60 ± 1.79	3.86 ± 3.85
Inflammation Grade	1.42 ± 0.63	1.37 ± 0.59	1.00 ± 0.41^#^	1.38 ± 0.51^&^
Fibrosis Stage	1.38 ± 1.14	1.60 ± 1.05	0.85 ± 0.69	1.31 ± 0.85
HBeAg (Positive)!	33 (41.77%)	25 (31.65%)	7 (53.85%)	3 (23.08%)

*P < 0.05 versus the value of the baseline level of group A

^&^*P* < 0.05 versus the value of the baseline level of group B

^#^*P* < 0.05 versus the value of the baseline level of group A

^@^*P* < 0.05 versus the value of 12 months later of group A

! no. (%)

### Pathological outcome

Pathological analysis indicated that the inflammation in patients of group B was lower than that in group A in the baseline. However, the grading of inflammation in group A showed decline after 12-month antiviral treatment. In group B, significant increase was observed in the inflammation grading after the 12-month follow-up compared with that of group A (*P* < 0.05). The fibrosis in cases of group A and group B showed progression. Additionally, no statistical differences were observed between the two groups (*P* > 0.05, Table 2).

For the cases with stable conditions, there were no significant differences in stability rate between group A and group B (*P* > 0.05). Whereas, the percentage of cases with improvement in disease conditions in group A was higher than that in group B. Meanwhile, in group A, 19 (24.05%) showed improvement in the inflammation and 18 (22.78%) showed improvement in fibrosis. In group B, 2 cases (15.39%) showed no improvement in the inflammation grade and the fibrosis stage. The disease progression rate was lower in group A compared with that of group B. The progression rate of inflammation grade in group A was significantly lower than that of group B (15.19% vs.38.46%, *P* < 0.05). In contrast, the progression rate of fibrosis stage in group A showed no statistical differences compared with that of group B (37.97% vs. 46.15%, *P* > 0.05). These suggested that patients in group A showed better response to antiviral therapy (Table 3).

**Table 3:** Degree of inflammation grading and fibrosis grading one year later

Pathological judgment	Group A (79, 85.87%)		Group B (13, 14.13%)	
	Inflammation grade	Fibrosis stage	Inflammation grade	Fibrosis stage
Stable condition	48, 60.76%	31, 39.24%	8, 61.54%	5, 38.46%
Improved condition	19, 24.05%	18, 22.78%	0, 0%	2, 15.39%
Progression condition	12, 15.19%	30, 37.97%	5, 38.46%^a^	6, 46.15%

^a^*P* < 0.05 versus the inflammation grade of group A; the data were presented as n, percentage

### Statistical analysis of the two groups before and after one year follow-up and the indices of the disease progression

For the comparison of progression condition indicators of inflammation grade, significant differences were observed in the baseline AFP levels in group A compared with that of group B (15.19% vs. 38.46%, *P* < 0.05). Additionally, we compared the indices of aggravation of fibrosis stage between the two groups. The HBV DNA between the two groups showed significant differences (*P* < 0.01). No significant differences were observed between the indicators in the group A or in the group B in the baseline level and after the 12-month follow-up. There was no statistically significant difference in the statistical analysis of the differences between the indicators in group A and group B in the 12-month follow-up.

## Discussion

HBV DNA serves as an indicator for HBV replication. Quantitative detection of HBV DNA is used as the main method for the monitoring and prognosis evaluation after antiviral efficacy. Generally, the virus activity in HBV DNA-negative patients is usually under control, and then their conditions are stable in a certain period. However, there are indeed possibilities of relapse under low immunity or stress due to the presence of cccDNA in liver tissues [12]. Nowadays, there are also disputes on the recommendation of antiviral therapy to HBV DNA-negative patients. Besides, most of the HBV DNA-negative patients are not appropriately treated, which may lead to progression several years after treatment [13]. Thus, special attention should be paid to monitor the alternations of liver of HBV DNA-negative patients by serology in clinical settings. In this study, 92 HBV DNA-negative hepatitis B patients who have been receiving antiviral therapy over 6 months were included in our study. In this study, we determined the effects of antiviral treatment on the prevention and prognosis of HBV reactivation in these patients and how to monitor the treatment response of patients with CHB under Chinese conditions.

Extensive studies have confirmed that antivital therapy contributes to prognosis of HBV DNA-positive hepatocarcinoma (HCC) patients [9, 10]. Besides, patients with latent HBV infection are apt to develop HCC in those with HBV DNA rather than HBV surface antigen in serum [14]. This suggested that there might be a certain relationship between HBV infection and liver cancer [15]. Thus, antiviral therapy is necessary to control the progression of hepatitis. Meanwhile, HBV DNA examination can also provide an accurate option for therapy. In our study, pathological analysis indicated that the inflammation grades and fibrosis stages of group B patients were lower than those of group A at the baseline level. In the 12-month follow up, the inflammation grade and fibrosis stage in cases of group B showed improvement, especially the inflammation grade (*P* < 0.05). In group A, the inflammation grade and fibrosis stage in cases of group showed improvement. These results demonstrated that HBV DNA negative patients with hepatitis B showed better response to antiviral therapy compared with the counterpart that were HBV DNA positive. There were really some HBV DNA-negative hepatitis B patients with poor response after antiviral treatment. This may be related to the fact that HBV is in a high level of replication and the condition is still progressing. Additionally, this may be related to the weak response of the body to liver inflammation and poor liver reserve.

ALT and AST were selected as liver function indicators in this study as they could reflect the liver damage specifically. A higher AST level exceeding ALT indicates deterioration in the hepatic parenchymal damage, which serves as a sign of chronic aggravation. Our results showed that AST and ALT levels in group B were higher than those in group A at the baseline level and one year after treatment, especially the ALT levels with statistical differences. In group A, the ALT level 12 months after treatment was lower than the baseline level. About 12 months after treatment, the liver function indices in group B were higher than the baseline level. These indicators may have a warning effect on the disease progression. In the presence of inflammation in liver, there might be aberrant changes in the liver synthesis and metabolism [16]. Patients with hepatitis B showed impaired liver function, and then the conversion between indirect bilirubin and direct bilirubin was hampered, which was manifested as elevation of indirect bilirubin and decline of direct bilirubin. In this study, TB and DB levels in group B were significantly lower than those in group A in the baseline levels (*P* < 0.05). Meanwhile, the DBIL level in cases of group A was significantly higher than that of group B in the baseline level. Furthermore, there were statistical differences in group A in terms of ALB, PT and PTA in the 12-month follow-up compared with the baseline level (*P* < 0.05). The PTA level of group B was significantly increased in the 12-month follow-up than the baseline level (*P* < 0.05). These findings suggested that the liver function in cases of group A was better than that of group B before the follow-up. In the 12-month follow-up, the liver function showed improvement in group A, and liver inflammation in group B was more obvious. AFP is a glycoprotein belongs to the albumin family,which does not express or underexpress on normal liver tissues, but the AFP level was increased significantly on hepatitis or cirrhosis tissues and the highest expression on liver cancer tissues [17]. It is mainly utilized as a serum marker for the diagnosis and efficacy monitoring of primary liver cancer. HBV infection is closely related to the high expression of AFP [18]. Our data showed that the AFP level in group A was significantly lower in the 12-month follow up than the baseline level (*P* < 0.05). The AFP level in patients of group B was increased at month 12, however, no statistical difference was identified. The AFP level of group A was higher at the baseline level than that of group B, and the AFP level of group A was lower at month 12 than that of group B. These results indicated that the improvement of liver inflammation in group A was better than that of group B. On this basis, we implied that the use of antiviral drugs and the sustained low level of HBV DNA may contribute to the improvement of hepatic function and prevention of disease progression. Taken together, the combined monitoring of liver function, PTA and AFP may be helpful to predict the progression of the disease. The improvement of inflammation in group A was superior to that of group B. This indicated the application of anti-viral agents and persistent low level of HBV-DNA may contribute to the improvement of liver function, which then delayed the disease progression.

To our best knowledge, some serum indicators are not adequate to prove the stage and extent of the liver damage. For example, in cases with poor liver conditions, hepatocyte necrosis is severe or the HBV is inactive, and then the inflammatory response is weak. The serological indicators suggest that the liver damage is weak. Therefore, many hepatitis B patients are not diagnosed until the end stage of hepatitis, especially HBV DNA negative patients, which may miss the best therapy period. The most direct evidence for clinical diagnosis of hepatitis is liver biopsy [19, 20]. HBV DNA is an important factor in the progression of HBV-associated liver disease, but there are indeed many limitations on the study of liver tissues, such as ethical controversy and complexity of sample collection [21]. Therefore, few reports investigated the relationship between the antiviral effects and the pathological grade, prognosis and disease progression of liver tissue in HBV DNA-negative hepatitis B patients. In this study, the degree of disease stability between group A and group B showed no statistical differences after 12-month antiviral therapy. Nevertheless, the rate of disease improvement in group B was lower than that in group A. The rate of disease progression in group B was higher than that of group A. This suggested that HBV DNA negative can not be used as a basis for the termination of HBV replication. High HBV DNA level indicated that the course of chronic hepatitis B patients may further progress to liver cirrhosis or even liver cancer. On one hand, this may be related to the mutations on the pre-C region of HBV, which resulted in decrease or elimination of HBeAg, viral gene mutations do not affect HBV replication, which then resulted in persistent infection of HBV [22]. Our results showed that the proportion of HBeAg positive in the case group were also reduced after continued anti-viral therapy. On the other hand, it may also be related to the continuous antiviral therapy in hepatitis B patients, which may cause resistance to certain drugs and subsequent recurrence of viral replication. These confirmed that there was a close relationship between HBV replication and disease progression. In future, we will focus on the specific mechanism in this process.

In summary, antiviral therapy is effective for treating HBV DNA-negative hepatitis B patients, which can improve anti-viral response. It contributes to the improvement of hepatic function and delay of disease progression. Great attention should be paid to the hepatitis B patients with significant fluctuation in the HBV DNA viral load after antiviral therapy as their disease conditions are still progressing. Close monitoring of liver function, PTA and AFP may help to find the change in the disease condition timely. In addition, it is necessary to suspect whether drug-resistant mutations occur in these patients. Besides, attention should be paid to determine the genotyping and drug-resistant mutations of hepatitis B virus. This also provides a new perspective to investigate the pathogenesis and efficacy of HBV-related disease progression. Further studies on the relationship between HBV DNA and HBV-related liver disease progression will be beneficial to the treatment and prevention of HBV-related liver diseases.

## Statement on human and animal rights

All human and animal studies have been approved by the appropriate ethics committee and have therefore been performed in accordance with the ethical standards laid down in the 1964 Declaration of Helsinki and its later amendments.

## Abbreviations

AFP: Alpha fetoprotein
ALB: Albumin
ALP: Alkaline phosphatase
ALT: Alanine aminotransferase
anti-HBc: antibodies against hepatitis B core antigen
anti-HBe: antibodies against hepatitis B e antigen
anti-HBs: antibodies against hepatitis B surface antigen
AST: Aspartate aminotransferase
DB: Direct bilirubin
GLB: Globulin
HBeAg: Hepatitis B e antigen
HBsAg: Hepatitis B surface antigen
HBV: Hepatitis B virus
PLT: Platelet count
PT: Prothrombin time
PTA: Prothrombin time activity
TB: Total bilirubin
WBC: White blood cell
γ-GT: Glutamyl transpeptidase
